# Basal and stimulated calcitonin for the diagnosis of medullary thyroid cancer: updated thresholds and safety assessment

**DOI:** 10.1007/s40618-020-01356-9

**Published:** 2020-07-12

**Authors:** L. Fugazzola, M. Di Stefano, S. Censi, A. Repaci, C. Colombo, F. Grimaldi, F. Magri, U. Pagotto, M. Iacobone, L. Persani, C. Mian

**Affiliations:** 1 Division of Endocrine and Metabolic Diseases, Istituto Auxologico Italiano IRCCS, Piazzale Brescia, 20, 20149 Milan, Italy; 2 Department of Pathophysiology and Transplantation, University of Milan, 20122 Milan, Italy; 3 Endocrinology Unit, Department of Medicine (DIMED), University of Padua, 35122 Padua, Italy; 4 Department of Medical and Surgical Sciences, Endocrinology, Prevention and Care of Diabetes Unit, Alma Mater Studiorum University of Bologna, Policlinic S. Orsola, Bologna, Italy; 5 Endocrinology, Metabolism and Clinical Nutrition Unit, University-Hospital of Udine, Udine, Italy; 6 Unit of Internal Medicine and Endocrinology, Laboratory for Endocrine Disruptors, Department of Internal Medicine and Therapeutics, Istituti Clinici Scientifici Maugeri IRCCS, and Department of Internal Medicine and Medical Therapy, University of Pavia, Pavia, Italy; 7 Endocrine Surgery Unit, Department of Surgical, Oncological and Gastroenterological Sciences (DiSCOG), University of Padua, 35122 Padua, Italy; 8 Department of Clinical Sciences and Community Health, University of Milan, 20122 Milan, Italy

**Keywords:** Calcitonin, Calcium test, Cut-off, Medullary thyroid cancer, Bradycardia

## Abstract

**Purpose:**

Reliable cut-offs for basal (bCT) and calcium stimulated calcitonin (casCT) are needed for an early and accurate diagnosis of medullary thyroid cancer (MTC).

**Patients and methods:**

Fifty-four new patients with nodular goiter were enrolled and analysed together with those previously published by our group for a total of 135 cases. bCT and casCT were measured by a highly sensitive method and the results compared with histological findings. In a subgroup of patients, cardiac rhythm was recorded before and during the calcium test.

**Results:**

In both females (F) and males (M), there was a significant correlation between tumor size and bCT levels (*P *< 0.001). The receiver operating characteristic plot analyses showed that, for bCT, the new cut-off points able to separate non-MTC from MTC patients were > 30 (F) and > 34 pg/mL (M), whereas the best casCT thresholds were > 79 (F) and > 466 pg/mL (M). bCT was shown to harbour a high accuracy, though some cases were diagnosed only upon stimulation test. Importantly, combining bCT, below or above the cut-offs, with casCT above the cut-offs, all the MTC cases were correctly identified. A reversible sinus bradycardia was observed in 9% of cases during the test.

**Conclusions:**

Refined cut-offs for bCT and casCT in patients with nodular goiter are reported. Sensitive bCT was shown to have a high accuracy, but the combination with casCT data was needed to identify all MTC cases. The reliability and safety of calcium test strongly favour the routine use of CT determination in nodular thyroid disease.

**Electronic supplementary material:**

The online version of this article (10.1007/s40618-020-01356-9) contains supplementary material, which is available to authorized users.

## Introduction

The routine measurement of serum calcitonin (CT) in patients with nodular/multinodular goiter represents the best method to precociously identify non-clinically evident MTC, very often in an early stage of progression, with a consequent positive impact on prognosis [1]. Although its cost effectiveness has been clearly demonstrated [2], the indication for the routine assessment of CT is still not universally shared and suggested by the scientific societies. In particular, though CT was recommended in the initial work-up of thyroid nodules by the European Consensus published in 2006 [3], more recent ATA and AACE/ACE/AME guidelines do not recommend either for or against routine measurement of serum CT (ATA, AACE) [4, 5] or limit the indication to patients to be submitted to surgery [6]. A very recent systematic review confirms the high sensitivity and specificity of CT testing, still questioning the value of its routine use due to the low prevalence of medullary thyroid cancer (MTC) (0.32% in thyroid nodular disease patients), and to the risk of overdiagnosis of possibly indolent tumors [7]. Nevertheless, since lymph nodal metastases are already present in up to 43% of MTCs ≤ 10 mm and 20% of them fail to be cured [8], the final goal of CT screening is the identification of micro-MTCs. Another reported drawback for the routine CT testing is related to the variability of CT measurement, depending on the laboratory/assay type [9, 10] and to the frequent finding of basal CT levels slightly higher than the normal range, which imply the need to perform a confirmatory stimulation test. The identification of universal and reliable cut-offs, which are still lacking for both basal and calcium stimulated CT (bCT and casCT), will likely support a more accurate diagnostic work up of thyroid nodules. In this context, when calcium (ca) test became the gold standard to evaluate casCt, we performed receiver operating characteristic (ROC) plot analyses showing that the best thresholds for the identification of MTC were > 26 and > 68 for bCT and > 79 and > 544 pg/mL for casCT in females (F) and males (M), respectively [11]. Since then, though these thresholds are currently used in the clinical practice, scanty data have been published. Data on bCT thresholds (> 35 pg/mL for F and > 46 pg/mL for M) have been recently reported to distinguish between non-MTC and MTC cases, though four different CT assay were used, all with different functional sensitivities [12]. In 2018, Niederle’s group reported that, in a series of 62 cases, MTC was predicted in F for bCT > 23 pg/mL or casCT > 780 pg/mL and in M for bCT > 43 pg/mL or casCT > 1500 pg/mL [13]. In 2020, the same Authors found in a larger series that these cut-offs were able to distinguish between a group of patients with definitive diagnosis of MTC and another group including C-cells hyperplasia and microMTC, and reported new cut-offs associated with lateral neck lymph node metastases [14].

Aim of this study was to identify cut-offs able to clearly differentiate between non-MTC and MTC cases in a large series of patients, followed by the same protocols in different Italian Institutions. The identification of reliable cut-offs is predicted to make CT screening more widely included in the initial diagnostic evaluation of thyroid nodules, particularly considering that ultrasonographic (US) features unique to this cancer are lacking. In this context, a large systematic review reported that US patterns of suspicion performed well even in MTC, though extremely specific signs used for the identification of papillary thyroid cancers, such as the taller than wide shape and the microcalcifications, were present in only 11% and 32% of MTCs, respectively [15]. Finally, since one of the hypothesized drawbacks of calcium test is related to a possible effect on cardiac function as reported in one patient [16] and not further confirmed, we recorded all cardiac variations during the test in a subgroup of the present series.

## Patients and methods

A total of 54 new patients (33 F and 21 M) affected with uni- or multi-nodular goiter were enrolled, to join their data, for the establishment of more precise cut-offs, with those (*n* = 81) previously published by our group [11], for a total of 135 cases. Patients carriers of a *RET* germline mutation were excluded, to avoid the possible interference exerted by diffuse C cells hyperplasia in the determination of the cut-offs. Indeed, aim of the present study was to calculate the best cut-offs for the identification of MTC, being C cells hyperplasia (CCH) considered as a non-MTC result together with benign thyroid diseases (such as follicular adenomas, papillary thyroid cancers, nodular goiters). Patients came from five different Italian Centres (Milan, Padua, Bologna, Udine, and Pavia), all measuring CT using a 2-site automated chemiluminescent immunometric assay (Immulite2000; Siemens Diagnostics), with an analytical sensitivity of 2 pg/mL, and all performing the calcium test as previously described [11]. Briefly, calcium gluconate was administered iv at the dose of 25 mg (2.3 mg or 0.12 mEq of elemental calcium)/kg, and the adjusted body weight was calculated (www.manuelsweb.com/IBW.htmforidealbodyweightandadjustedbodyweightcalculator) for each patient to avoid overdosage. After a basal blood sampling for CT, calcium gluconate was administered iv at 5 mL/min, with a minimum time of administration of 3 min. All the possible causes interfering with the correct CT assessment were excluded and drugs, such as proton-pump inhibitors, withdrawn for 2 weeks. All patients were submitted to ultrasound (US) evaluation and, when appropriate, to cytology.

Patients with CT levels higher than the normal range were submitted to at least an additional control sampling by the above reported assay, and only those with confirmed CT levels above the cut-off were included. Patients enrolled in the present study had nodular/multinodular goiter (MNG) with bCT levels ≥ 10 pg/mL (30 F and 20 M), and underwent surgery according to a surgical and pathological protocol based on International guidelines [9], and shared among the five participating Centers (Table 1). Four patients with bCT levels detectable but < 10 pg/mL (6–9 pg/mL), with huge MNG or suspicious cytology were also enrolled. All cases with a suspicion of CCH/MTC based on previous cut-offs for basal and/or stimulated CT (49/54 cases) were submitted to total thyroidectomy and prophylactic bilateral central neck dissection, whereas the remaining five patients were submitted to total thyroidectomy alone, for tracheal compression and/or for cytological suspicion (indeterminate or suspicious for papillary thyroid cancer). To note, among the 81 patients previously reported, 50 had a bCT ≥ 10 pg/mL and the remaining had a bCT < 10 pg/mL and were submitted to surgery for reasons other than a suspicion of MTC [11]. Patients with thyroid autoimmunity were included both in the previous [11] and in the present study (*n* = 9 in the previous, *n* = 7 in the present), since autoimmunity has been demonstrated not to influence CT levels [17].

**Table 1:** Clinical features of the patients firstly described in the present study

ID/Age, years	Origin	Ct pre-Tx, pg/ml		Histology (mm)	TNM*	CCH
		Basal	Peak			
*Females*						
C.D./46	#1	6.1	161	MNG	–	No
P.S./51	#1	8.9	188	PTC (3)	pT1aNx	No
B.G./48	#5	9	210	PTC (n.a.)	pT1aNx	No
G.M.A./67	#3	11.6	118	MTC (7)	pT1aN0	No
P.N./51	#3	11.9	162	MTC (3)	pT1aN0	No
D.N./50	#2	126	175	MTC (12)	pT1bN1a	No
F.F./55	#5	126.2	181	MTC (9)	pT1aN0	Yes
T.M.G./52	#1	11.6	188	MNG	–	Yes
L.R.R/50	#1	50.4	208	PTC(8) + MTC (6)	pT1N0/pT1aN0	No
V.T./73	#4	12.9	216	MTC (13)	pT1bN0	No
B.E./52	#5	60.5	222	MNG	–	Yes
R.M./57	#1	15.7	279	MNG	–	Yes
V.M./64	#1	28	296	MNG	–	Yes
P.R./38	#3	24.7	317	MNG	–	Yes
S.A./70	#3	20.3	379	MTC (3)	pT1aN0	No
L.L./70	#4	77.9	430	MTC (10)	pT1aN1a	No
T.V./67	#3	376	461	MNG	–	Yes
B.L./50	#3	30	487	PTC (10)	pT1aNx	Yes
C.C./58	#2	39.3	488	MTC (11)	pT1bN0	No
M.A./64	#1	17.3	614	FA	–	Yes
G.M.L./47	#1	11.6	623	MNG	–	Yes
S.L./54	#3	16.9	666	MNG	–	Yes
R.V./71	#3	54	678	MTC (4)	pT1aN0	No
Z.A./62	#2	91.5	736	MTC (12)	pT1bN0	No
C.L./59	#3	28.9	891	MNG	–	Yes
B.M.G./64	#2	49.2	974	MTC (12)	pT1bN0	No
R.M./66	#1	89.7	998	MTC (11)	pT1bN0	No
P.A./52	#2	25.8	1010	MTC (6)	pT1aN0	No
F.L./82	#1	462	1236	MTC (12)	pT1bN0	Yes
P.R./68	#1	685	1297	MTC (11)	pT1bN1a	No
G.G/69	#1	80	1991	MTC (7)	pT1aN0	No
G.B./64	#1	89	2707	MTC (7)	pT1aN0	Yes
F.B./58	#2	64	9760	MTC (9)	pT1aN0	No
*Males*						
S.W./54	#2	7.6	84.7	MNG	–	Yes
D.I./49	#2	11.8	147	PTC (1.5–2)	pT1amNx	Yes
S.E./56	#2	12.9	169	MNG	–	n.a
F.G./64	#4	37.5	169	MTC (8)	pT1aN1a	No
F.P./42	#3	13	187	PTC (40)	pT3aN1a	n.a
R.B./68	#1	14.1	201.6	MNG	–	Yes
B.R./68	#2	12.3	206	MNG	–	Yes
G.B./58	#4	23	211	MTC (18)	pT1bN1a	No
C.R./66	#3	14.5	238	PTC (3)	pT1aNx	Yes
P.G./58	#2	19.4	238	MNG	–	Yes
C.R./55	#5	28	277	PTC (n.a.)	pT1aNx	No
B.F./45	#3	20.6	462	MNG	–	No
R.M./42	#3	34	466	PTC (7)	pT1aN0	No
N.A./72	#3	62.3	555	MNG	–	Yes
M.C./69	#4	193	580	MTC (11)	pT1bN1a	No
P.E./54	#1	23.8	665	MTC (16)	pT1bN0	No
G.P./57	#1	18.3	706	PTC (5)	pT1aNx	Yes
P.A./46	#2	27.7	1020	MTC (17)	pT1bN0	No
B.R./52	#2	472	1140	MTC (15)	pT1bN1b	No
M.M./66	#1	47	1357	MTC (12)	pT1bN0	No
B.M.V./35	#1	126	1484	MTC (9)	pT1aN0	No

#1 Milan; #2 Padua; #3 Bologna; #4 Udine; #5 Pavia; *Tx* total thyroidectomy, *MNG* multinodular goiter, *PTC* papillary thyroid cancer, *MTC* medullary thyroid cancer, *FA* follicular adenoma, *CCH* C cells hyperplasia; * TNM staging AJCC UICC 8th edition; n.a.: not available

To better evaluate the side effects of calcium test, we examined 133 consecutive cases (74 F and 59 M), submitted to this procedure in the Milan site during 5 years (2014–2018). Moreover, in 67 cases (43 F and 24 M), which underwent calcium test in the last 22 months (April 2018–January 2020), we evaluated data coming from the continuous ECG evaluation pre-, during and post-test.

All patients gave their informed consent to be submitted to the test and to include the results obtained in the present study, which has been approved by the Ethical Committees of the Institutions involved.

### Statistical analyses

The correlation between MTC diameter, bCT levels and casCT, and whiskers plot graphs were done by means of a regression analysis and Chi-square test, respectively.

The gender-specific cut-off levels corresponding to the highest accuracy to differentiate between non-MTC and MTC cases were assessed by receiver-operating characteristic (ROC) curves, and their sensitivity and specificity were defined. It is worth noting that the data obtained in the present series were pooled with those already reported by our group [4] to increase the potency of the analysis. In a following step, the obtained cut-offs were used to evaluate if the combination of bCT and CasCT cut-offs could improve the prediction of MTC. Statistical significance was defined as *P* < 0.05. All statistical analyses were performed using SPSS version 8.0 for Windows and MedCalc Software version 11.6.1 for Windows.

## Results

The histological examination showed a MTC in 27/54 (50%) cases, 19/33 (57.6%) females and 8/21 males (38%). All MTCs were pT1 (pT1a in 13 and pT1b in 14 cases), and the majority of them had no regional lymph nodes involved (p N0 in 20 cases and pN1 in 7, i.e., 26% of cases). No patient had distant metastases. In the remaining cases, histology showed a MNG in 10 F and 7 M (associated with CCH in nine cases), a follicular adenoma in 1 F (with CCH), a PTC in 4 F (associated with MTC in one case) and in 6 M, associated with CCH in four cases (Table 1). The US features and the cytological results, when available, of the 27 patients with a final diagnosis of MTC are reported in Table 2. Interestingly, according to the Italian classification of thyroid cytology [18], the majority of the nodules submitted to biopsy were classified as TIR1 (22.2%) or TIR2 (44.4%), while the remaining 33.3% were equally divided into TIR3B, TIR4, and TIR5 cases. At US, the majority of nodules were solid and hypoechoic (77.7 and 76.9%, respectively), while a minority were mixed or spongiform; regular margins were recorded in 64% of cases and microcalcifications were present in 38.5% of nodules. Finally, the majority of nodules were round (65%), while a taller than wide shape was found in only 10% of patients.

**Table 2:** Ultrasonographic features and cytological results of the 27 patients with a final diagnosis of MTC

ID/years	Origin	Cytology	UNG/MNG	Suspicious nodule (lobe/mm)	US features					MTC size at histology (mm)
					Composition	Echogenicity	Margins	Micro-calcifications	Shape	
G.M.A./67	#3	TIR1	MNG	Left/7	Solid	Hypoechoic	Regular	No	Round	7
P.N./51	#3	TIR1	MNG	Left/5	Mixed	Hypoechoic	Regular	Yes	Round	3
N.D./50	#2	n.a	MNG	Right/12	Solid	Hypoechoic	Irregular	Yes	Round	12
F.F./55	#5	n.a	UNG	Right/10	Solid	Hypoechoic	Regular	No	Round	9
L.R.R./50	#1	n.a	MNG	Right/7	Mixed	Hypoechoic	Regular	Yes	Round	6
V.T./73	#4	TIR2	UNG	Left/13	Solid	Hypoechoic	Irregular	No	Round	13
S.A./70	#3	TIR2	MNG	Left/6	Solid	Isoechoic	Regular	No	n.a	3
L.L./70	#4	TIR4	UNG	Right/7	Solid	Hypoechoic	Irregular	No	Round	10
C.C./58	#2	TIR3B	UNG	Left/9	Solid	Isoechoic	Irregular	Yes	Ovoid	11
R.V./71	#3	TIR1	MNG	Right/8	Solid	Hypoechoic	Regular	No	Round	4
Z.A./62	#2	n.a	GMN	Left/11	Solid	Hypoechoic	Regular	No	Round	12
B.M.G./64	#2	TIR1	UNG	Right/12	Solid	Hypoechoic	Irregular	No	Ovoid	12
R.M./66	#1	n.a	UNG	Right/16	Spongiform	Isoechoic	Regular	No	Ovoid	11
P.A./52	#2	TIR2	UNG	Left/8	Solid	Hypoechoic	Regular	Yes	Ovoid	6
F.L./82	#1	TIR2	MNG	Left/38	Mixed	Hypoechoic	Regular	No	Round	12
P.R./68	#1	n.a	MNG	Left/16	Solid	Isoechoic	Regular	Yes	n.a	11
G.G./69	#1	TIR2	MNG	Left/10	Solid	Hypoechoic	Regular	No	n.a	7
G.B./64	#1	n.a	MNG	Left/10	Solid	Hypoechoic	Irregular	No	Round	7
F.B./58	#2	n.a	GMN	Right/8	Solid	n.a	n.a	N.a	n.a	9
F.G./64	#4	TIR3B	UNG	Right/7	Solid	Hypoechoic	Irregular	No	Round	8
G.B./58	#4	TIR4	UNG	Left/19	Solid	Hypoechoic	Irregular	Yes	Taller than wide	18
M.C./69	#4	TIR5	UNG	Left/24	Mixed	Hypoechoic	Irregular	Yes	Taller than wide	11
P.E./54	#1	TIR5	MNG	Right/26	Spongiform	Isoechoic	Regular	No	n.a	16
P.A./46	#2	TIR2	UNG	Left/18	Solid	Hypoechoic	Regular	Yes	Ovoid	17
B.R./52	#2	TIR2	UNG	Right/12	Solid	Hypoechoic	n.a	Yes	n.a	15
M.M./66	#1	n.a	MNG	Right/28	Solid	Isoechoic	Regular	No	n.a	12
B.M.V./35	#1	TIR2	UNG	Left/10	Solid	Hypoechoic	Regular	No	Round	9

Cytological classification by Italian consensus for the classification and reporting of thyroid cytology (J Endocrinol Invest. 2014 37(6):593–9); n.a.: not available; *UNG* uni-nodular goiter, *MNG* multinodular goiter, *MTC* medullary thyroid cancer

The below reported results refer to the combination of these new cases with our previously published [11]. The whole series includes a total of 135 cases (77 F and 58 M), with 47 MTC cases (29 F and 18 M). In particular, MTCs < 5 mm were 3/27 in the new series and 1/20 in the previous one, those ≥ 5 < 10 mm were 9/27 in the new series and 7/20 in the old one.

### Correlation between bCT levels and tumour size

**Fig. 1:**
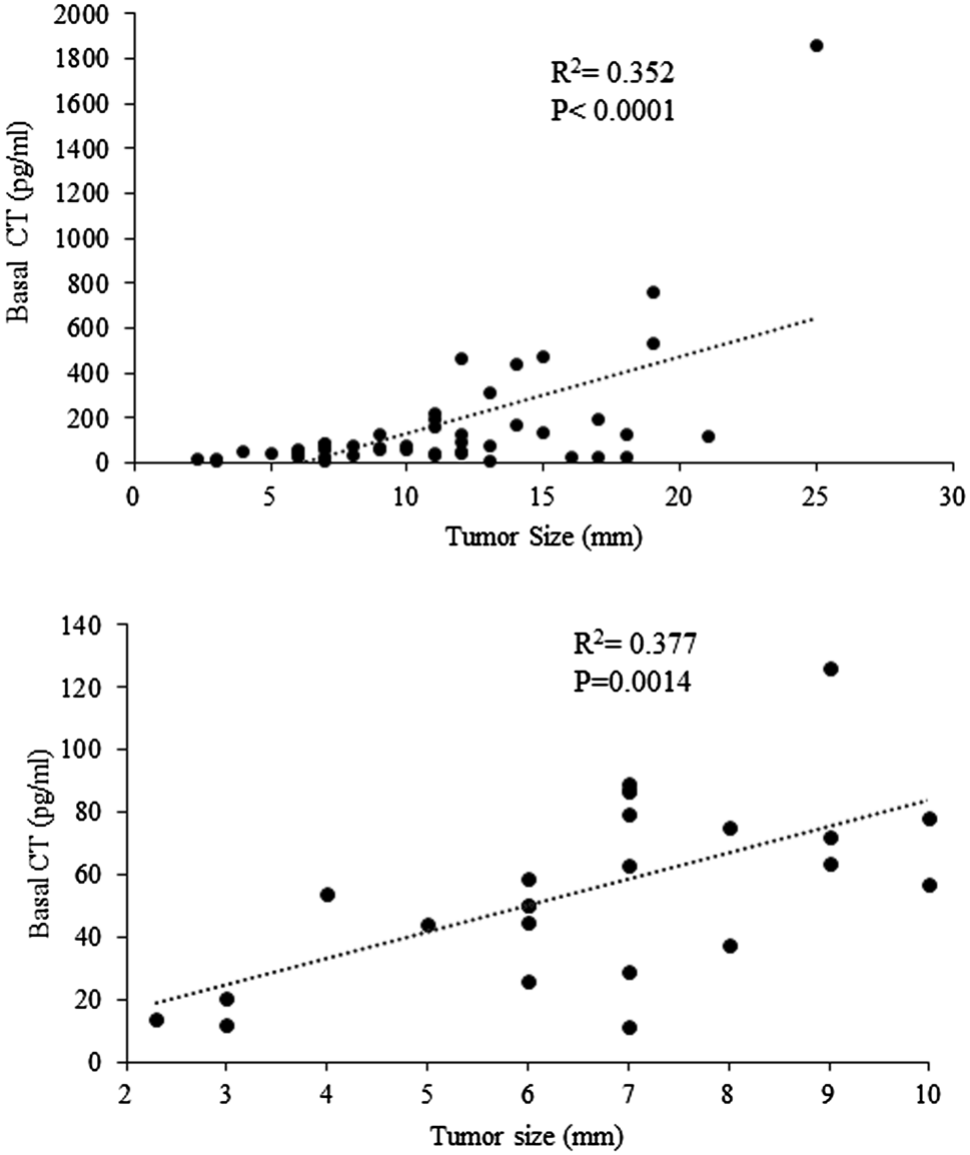
the highly significant correlation between basal CT levels and tumor size is shown (upper part); an outlier level (1860 pg/mL) is not represented in the whiskers plot for graphical reasons, but it was included in the statistical analysis. The significant correlation exists also considering only tumors ≤ 10 mm (lower part)

The mean and median CT levels were 21.38 and 15 pg/mL (range 2.8–53.7) for tumors < 5 mm, 52.26 and 58.8 pg/mL (range 5.6–126) for 5–10 mm tumors, 227.6 and 121 pg/mL (range 12.9–1860) for tumors ≥ 10 mm (*P* < 0.001) (data not shown). A strongly significant correlation between tumor size and bCT levels was found either considering all MTCs (*R*^2^ = 0.352, *P* < 0.0001) or only microMTCs (≤ 10 mm) (*R*^2^ = 0.377, *P* = 0.0014) (Fig. 1). On the other hand, the correlation between tumor size and casCT levels was low (*R*^2^ = 0.1213, *P* = 0.01) (data not shown).

### Sensitivity, specificity, positive and negative predictive value, and accuracy for MTC (ROC analysis)

**Fig. 2:**
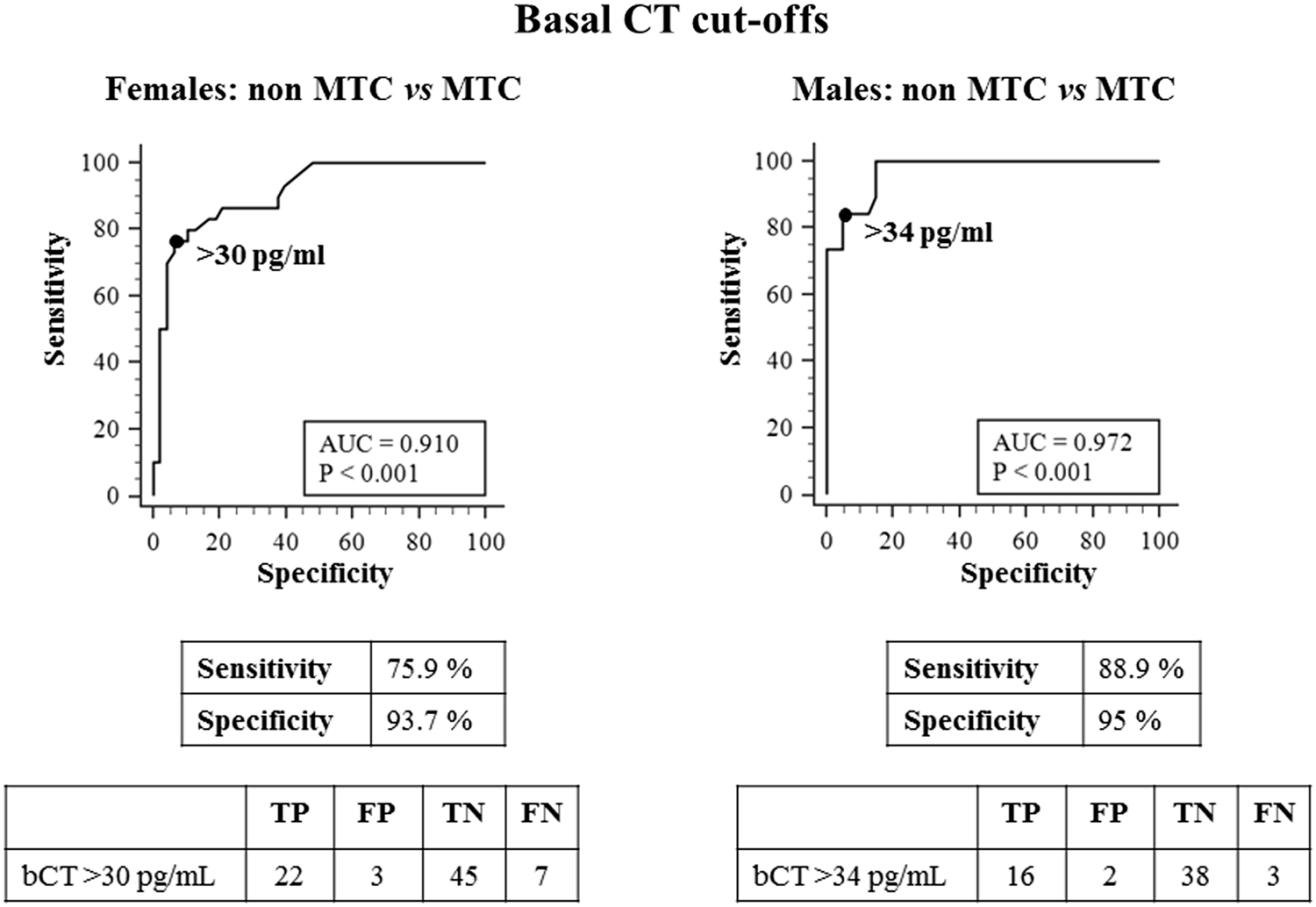
ROC curve analyses to identify the cut-off levels for basal CT with the highest accuracy to differentiate between non-MTC and MTC in females (left panel) and males (right panel). Non-MTC includes nodular goiter with and without C cells hyperplasia. Abbreviations: TP: true positive, FP: false positive, TN: true negative, FN: false negative cases

**Fig. 3:**
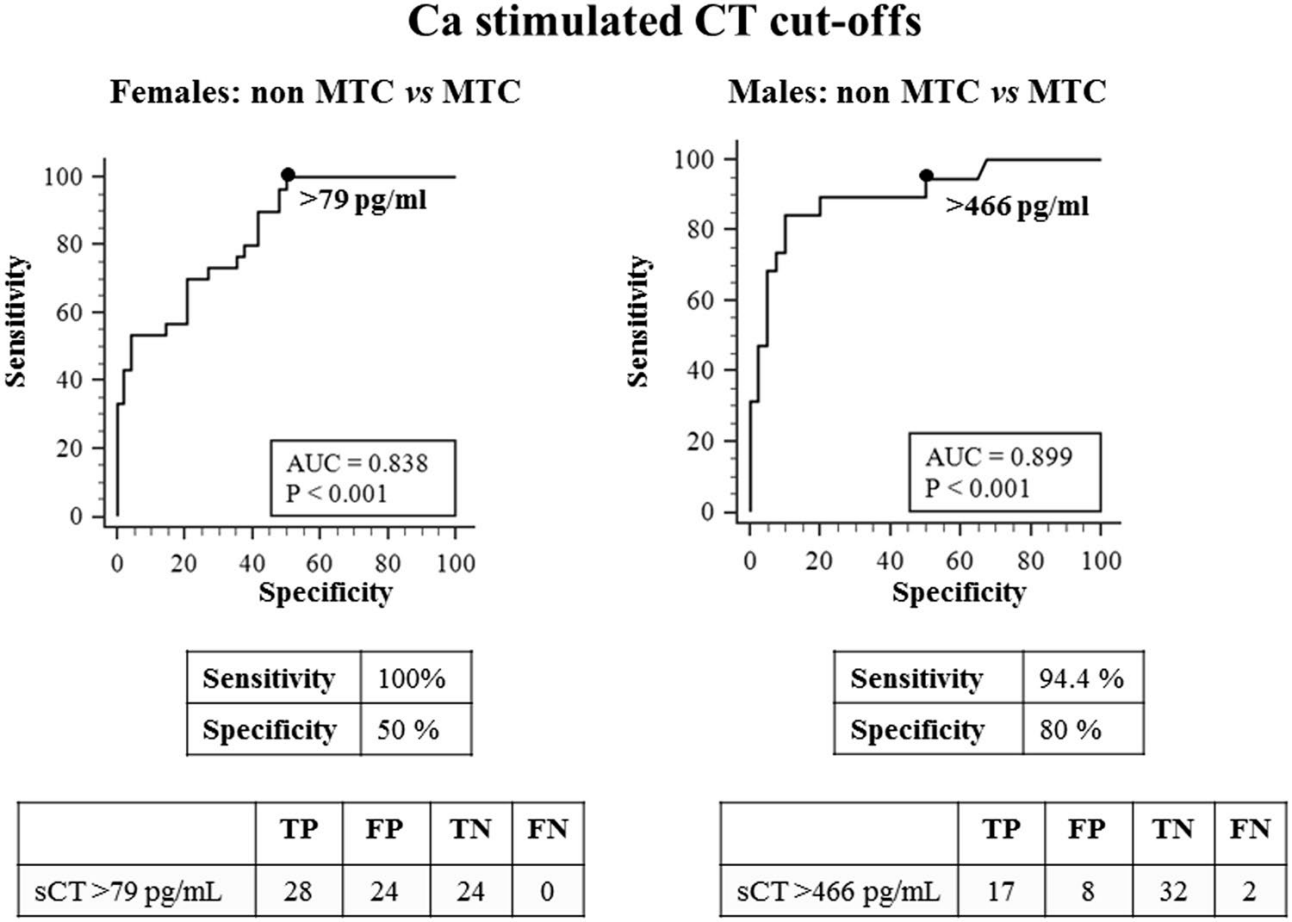
ROC curve analyses to identify the cut-off levels for calcium stimulated CT with the highest accuracy to differentiate between non-MTC and MTC in males (left panel) and males (right panel). Non-MTC includes nodular goiter with and without C cells hyperplasia. Abbreviations: TP: true positive, FP: false positive, TN: true negative, FN: false negative cases

ROC plot analyses were used to find the bCT and casCT thresholds able to differentiate between non-MTC (including nodular goiter associated or not with CCH) and MTC for women and men. The best thresholds for bCT were > 30 pg/mL and > 34 pg/mL, in F and M, respectively (Fig. 2). Interestingly, the same thresholds were obtained by ROC curves after the exclusion from the whole series of cases with bCT ≤ 10 pg/mL (23 F and 15 M) (data not shown). On the other hand, casCT > 79 and > 466 pg/mL, in F and M, respectively, were selected as the most accurate to distinguish non-MTC cases from patients with MTC (Fig. 3).

### Combined cut-offs

**Fig. 4:**
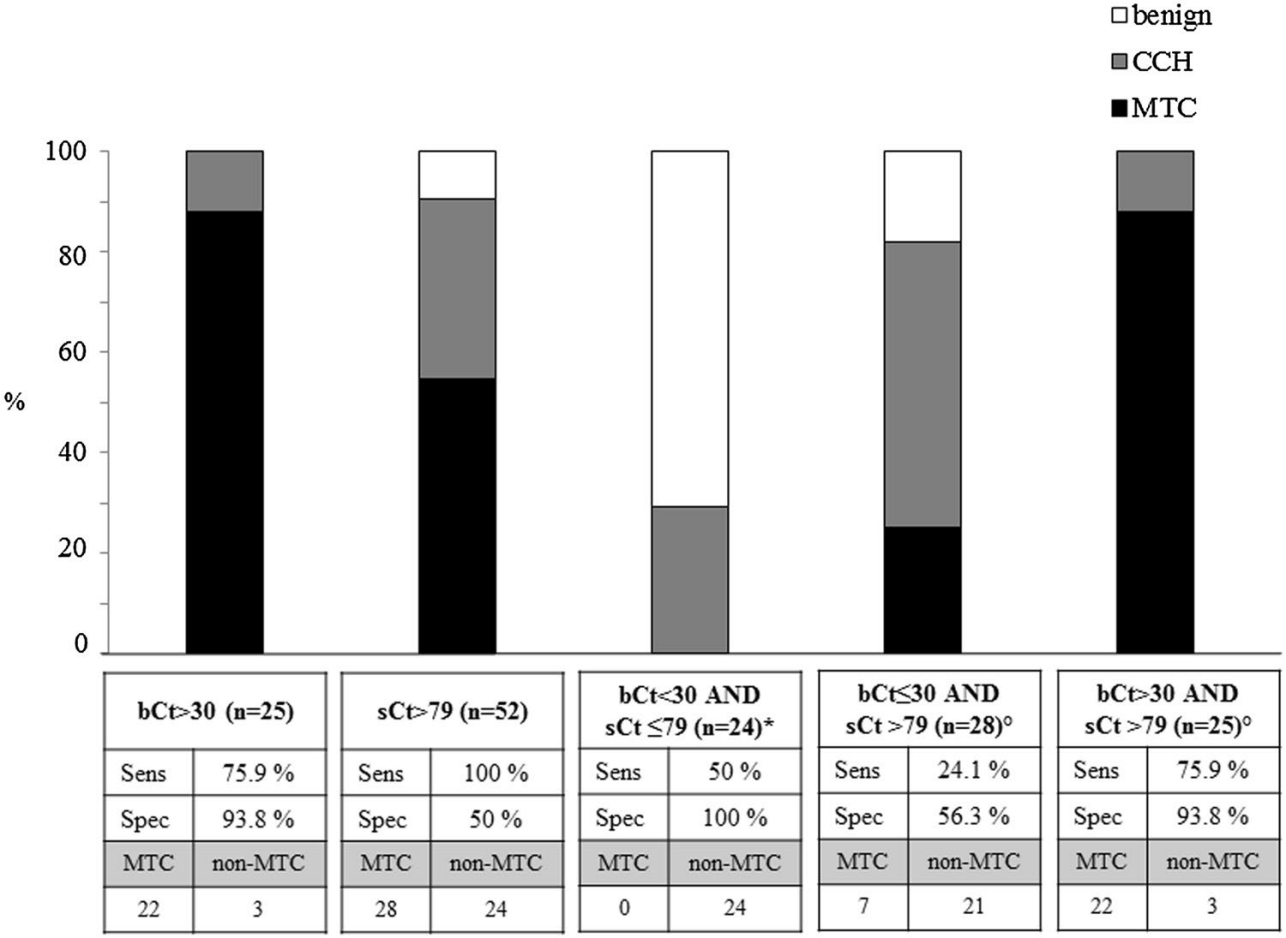
prevalence of MTC and goiter not associated (benign) or associated with C cells hyperplasia (CCH) by preoperative basal CT and calcium stimulated CT levels, alone or in combination, in female patients who underwent total thyroidectomy. * for the identification of non-MTC cases; ° for the identification of MTC cases

**Fig. 5:**
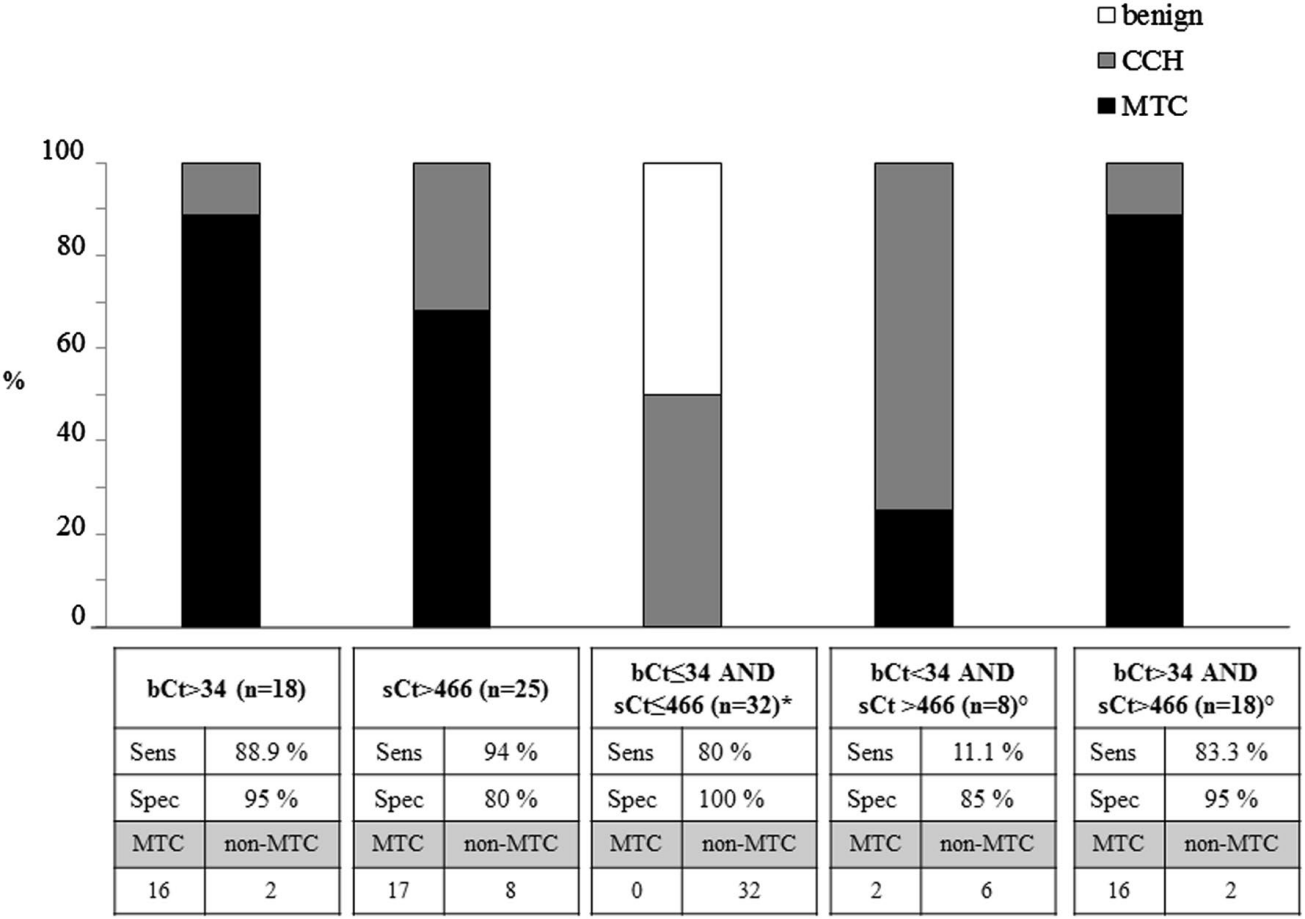
prevalence of MTC and goiter not associated (benign) or associated with C cells hyperplasia (CCH) by preoperative basal CT and calcium stimulated CT levels, alone or in combination, in male patients who underwent total thyroidectomy. * for the identification of non-MTC cases; ° for the identification of MTC cases. The number of patients with MTC or non-MTC are also reported

After the identification of the most accurate cut-offs, we aimed to verify if the combination of bCT and casCT could improve the diagnostic performance for MTC identification (Figs. 4, 5). Interestingly, all cases with both bCT and casCT levels below the thresholds for MTC determination were found at histology with a benign nodular diseases, associated with CCH in 29 and 50% of F and M cases, respectively. On the contrary, the majority (88 and 89% for females and males, respectively) of patients with bCT and casCT higher than the cut-offs established in the present study, had a MTC at histology. In both situations, a high specificity was obtained, ranging 94–100%. Since all patient with a bCT higher than the threshold had a casCT over the cut-off, but one male who had a bCT > 34 and CasCt < 466 pg/mL, the accuracy of bCT was almost superimposable to that found for bCT combined with CasCT.

Nevertheless, seven female MTCs (all pT1aN0, tumor size ranging 2.3–13 mm) and two male MTCs (all pT1bN0, tumor sizes 16 and 17 mm) would have been misdiagnosed by considering bCT over the threshold either alone or in combination with a casCT higher than the cut-off, since they had bCT below and casCT above the cut-off. Thus, the important finding of this study is that, by combining bCT below or above the cut-offs with casCT above the cut-offs, all the MTC cases were correctly identified, either in F and M patients.

### Prevalence of CCH and hypercalcitoninemia in non-MTC cases

We evaluated the prevalence of CCH in non-MTC cases, i.e., those with a final diagnosis of nodular goiter or PTC. In the whole series, CCH was found to associate with PTC in 45.7% of cases (6/18 F and 10/17 M), and with MNG in 73.8% of cases (17/25 F and 14/17 M) (*P* = 0.31). As far as CT levels concerns, in females, mean bCT ± SD levels were 19.12 ± 29.81 and 26.82 ± 68.54 pg/mL in patients with a final diagnosis of PTC and in those with a final diagnosis of MNG, respectively (*P* = 0.59), while casCT ± SD levels were 249.55 ± 545.31 for those with PTC and 264 ± 287 pg/mL for those with MNG (P = 0.9). In males, mean bCT ± SD levels were 17.65 ± 19.39 and 19.43 ± 18.63 pg/mL in patients with PTC and MNG, respectively (*P* = 0.77), while casCT ± SD levels were 335.2 ± 324.9 for those with PTC and 394.5 ± 434.2 pg/mL for those with MNG (*P* = 0.64).

### Evaluation of calcium test safety

To assess the safety of calcium test, in the subgroup of patients from Milan, all the adverse events were recorded during the test, and possible rhythm alterations were considered before and during the test (Supplemental Figure 1). The most frequent side effect recorded was the feeling of warmth (57% of cases), whereas other side effects were present in a minority of cases. Concerning the cardiological status of the patients before being submitted to calcium test, a sinus rhythm was recorded in the majority of cases (67.2%), a sinus bradycardia was present in 16.4% of patients, while other rhythm alterations were found in the minority of cases (1.5–4.5%). Interestingly, at the end of the test, the percentage of patients with a sinus rhythm significantly decreased (58.2%) due to an increase of patient with a sinus bradycardia (25.4%).

Finally, it is worth to note that the test was performed without side effects even in patients with hypertension (20%), with previous myocardial infarction (3.7%), with deep vein thrombosis, previous stroke, stenotic carotid artery, history of atrial fibrillation (0.7–3%).

## Discussion

The present study, including a novel multicentric series, provides data on the most accurate bCT and casCT cut-offs for the preoperative identification of subjects with MTC. These findings represent a refinement of those reported by our group in 2014 in a more limited number of cases [11]. After pooling data, for a total of 135 patients, ROC curve analyses were used to compare the preoperative bCT and casCT levels with the histological findings. The bCT cut-offs able to separate non-MTC from MTC patients were > 30 in F and > 34 pg/mL in M. Interestingly, a strong correlation was found between tumor size and bCT, either considering all tumors or limiting to micro-MTCs. The best CasCT thresholds for the identification of MTC were > 79 and > 466 pg/mL for women and men, respectively. The casCT thresholds are equal or similar to those previously reported (> 79 and > 544 pg/mL, F and M) [11], whereas present bCT cut-offs differ from the previous report (> 26 and > 68 pg/mL, F and M) [11], particularly in males.

After the establishment of these new cut-offs, we evaluated their diagnostic power either alone or in combination. Similar to our previous data [11] and a recent report [14], bCT was shown to have an accuracy superimposable to that derived from the combination of bCT and casCT, indicating that, in both F and M, bCT values are extremely good predictors of MTC, and suggesting that serum CT assays with improved functional sensitivity may avoid the stimulation test in several conditions. Nevertheless, in the present series, we recorded patients for whom MTC could be diagnosed only upon calcium test results.

Present cut-offs have been established to discriminate MTC cases from non-MTC cases (i.e., follicular adenomas, multinodular goiters, PTCs), independently from their possible association with CCH. Indeed, CCH was found to be highly prevalent in both goiters and PTC cases, without significant differences, and is responsible for the bCT levels above the normal range recorded in these cases. We previously demonstrated that CCH is frequent nearby benign or PTC nodules likely due to a paracrine effect on C cells [19]. Nevertheless, the real impact of PTC on bCT levels is still debated and has been poorly explored. In a study including almost 500 patients, no significant differences in the prevalence of basal hypercalcitoninemia was found between patients with or without PTC [17], but stimulated data were not available. In our whole series, we found no significant differences in either bCT or casCT levels between patients with PTC or benign diseases at histology. Thus, hypercalcitoninemia seems not to be indicative of PTC and, when found in patients with thyroid nodules, it should be regarded as being suggestive of MTC.

The cut-offs defined in the present series are different with respect to those recently reported by another group [13], particularly related to stimulated data, which are higher and that would have led to the loss of the majority of our MTC cases (data not shown). This is consistent with the lower sensitivity that Niederle et al. found for both bCT and casCT with respect to our findings [14]. Although this evidence shows that the direct comparison of results obtained in different laboratories is difficult, making it challenging to find universal CT cut-offs, a possible explanation of this discrepant data could reside in the different population examined, being the tumors with pT > 1 higher in Niederle’s than in our series (16 vs 2%). In this context, since lymph nodal metastases are already present in up to 43% of MTCs ≤ 10 mm [8], and the aim of the routine CT measurement is to perform an early surgical intervention to improve cure rates [20], we believe that our cut-offs are more appropriate to reach that goal. Moreover, we tried to overcome the problem of comparability by pooling data from different Institutions using the same assay. Since the sensitive method used in the present study is widely diffused, and the calcium test is now well standardized, present cut-offs are likely to be reliably used in many different Institutions. We propose a diagnostic and therapeutic decision based only on the acquirement of a bCT and a casCT. Due to the variability in CT measurements among commercial assays [9, 10], bCT should be assayed by a sensitive method, and repeated once in the absence of interfering factors. This strategy may be preferable and more cost effective than the basal re-evaluation at intervals of 3–6 months suggested for patients with bCT values falling in the grey zone [21].

The high tolerability of the Ca test was confirmed, with minor side effects mostly consisting of a brief feeling of warmth. The test was safely performed also in patients with previous diseases, such as myocardial infarction o stroke, or atrial fibrillation, and the only effect observed was the onset of sinus bradycardia in 9% of patients during the test, with a prompt recovery of the basal rate at the end of the procedure.

Finally, US evaluation showed that in our series MTC nodules are frequently solid and hypoechoic, with a round shape and regular margins, consistent with data reported in a large meta-analysis [15], and confirming that suspicious sonographic features are less often present in MTCs than in PTCs, with the exception of hypoechogenicity. Moreover, the low diagnostic accuracy of cytology in MTC [9, 10] has been confirmed in the present series, being the majority of our cases classified as inadequate or benign.

In conclusion, we report accurate cut-offs for basal and calcium stimulated CT in patients with nodular goiter for the clear separation between non-MTC and MTC cases. Although basal CT cut-offs have been shown to identify most cases, the combination with a casCT is needed, if the aim is to diagnose all MTCs. The reliability and safety of calcium test support the routine use of CT determination in nodular thyroid disease, particularly considering that MTCs do not present a unique US pattern and can be misdiagnosed at cytology.

## Electronic supplementary material

Below is the link to the electronic supplementary material.

**Supplemental Figure 1: **side effects recorded in 133 consecutive patients during calcium test (upper part). Cardiac rhythm pre-test and during the test in 67 patients submitted to continuous monitoring.1 (TIF 922 kb)
