# Supplementary figures and images for: Basal and stimulated calcitonin for the diagnosis of medullary thyroid cancer: updated thresholds and safety assessment
Source: J Endocrinol Invest. 2020 Jul 12;44(3):587–97. doi: 10.1007/s40618-020-01356-9 (PMC7878259; doi:10.1007/s40618-020-01356-9)

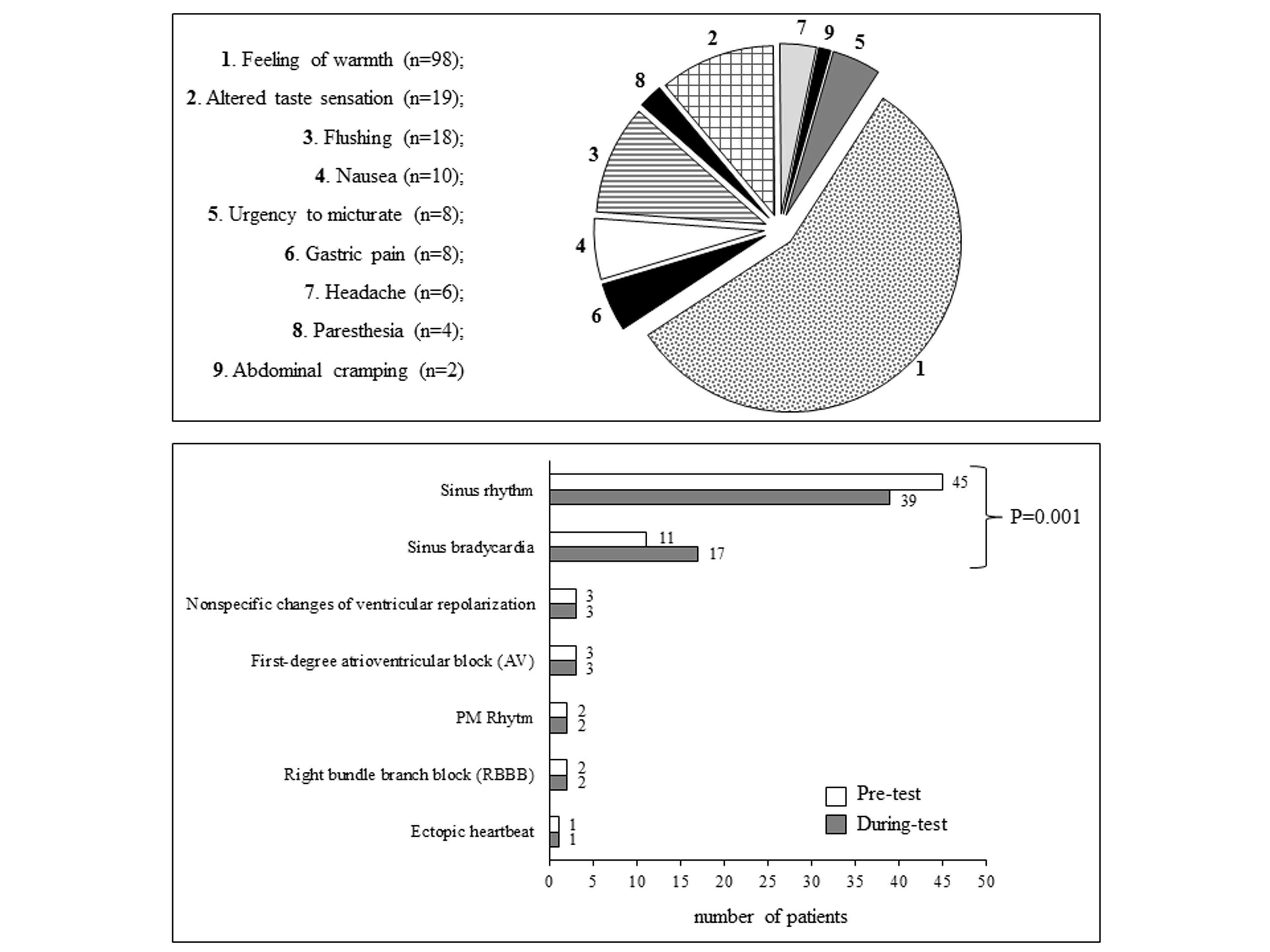

Supplement: Supplementary file 1 — Supplemental Figure 1: side effects recorded in 133 consecutive patients during calcium test (upper part). Cardiac rhythm pre-test and during the test in 67 patients submitted to continuous monitoring.1 (TIF 922 kb) [file 40618_2020_1356_MOESM1_ESM.tif]
